# Antibacterial Multi-Layered Nanocellulose-Based Patches Loaded with Dexpanthenol for Wound Healing Applications

**DOI:** 10.3390/nano10122469

**Published:** 2020-12-09

**Authors:** Daniela F. S. Fonseca, João P. F. Carvalho, Verónica Bastos, Helena Oliveira, Catarina Moreirinha, Adelaide Almeida, Armando J. D. Silvestre, Carla Vilela, Carmen S. R. Freire

**Affiliations:** 1 CICECO–Aveiro Institute of Materials, Department of Chemistry, University of Aveiro, 3810-193 Aveiro, Portugal; danielafonseca@ua.pt (D.F.S.F.); joao.pedro.carvalho@ua.pt (J.P.F.C.); catarina.fm@ua.pt (C.M.); armsil@ua.pt (A.J.D.S.); 2 Department of Biology and CESAM, University of Aveiro, 3810-193 Aveiro, Portugal; veronicabastos@ua.pt (V.B.); holiveira@ua.pt (H.O.); aalmeida@ua.pt (A.A.)

**Keywords:** oxidized bacterial cellulose, chitosan, alginate, layer-by-layer assembly, multi-layered patches, dexpanthenol, wound healing

## Abstract

Antibacterial multi-layered patches composed of an oxidized bacterial cellulose (OBC) membrane loaded with dexpanthenol (DEX) and coated with several chitosan (CH) and alginate (ALG) layers were fabricated by spin-assisted layer-by-layer (LbL) assembly. Four patches with a distinct number of layers (5, 11, 17, and 21) were prepared. These nanostructured multi-layered patches reveal a thermal stability up to 200 °C, high mechanical performance (Young’s modulus ≥ 4 GPa), and good moisture-uptake capacity (240–250%). Moreover, they inhibited the growth of the skin pathogen *Staphylococcus aureus* (3.2–log *CFU* mL^−1^ reduction) and were non-cytotoxic to human keratinocytes (HaCaT cells). The in vitro release profile of DEX was prolonged with the increasing number of layers, and the time-dependent data imply a diffusion/swelling-controlled drug release mechanism. In addition, the in vitro wound healing assay demonstrated a good cell migration capacity, headed to a complete gap closure after 24 h. These results certify the potential of these multi-layered polysaccharides-based patches toward their application in wound healing.

## 1. Introduction

Natural polymers are being explored as building blocks to engineer multifunctional sustainable materials with sophisticated architectures via biomacromolecular assembly strategies. Among the prevailing methodologies to design such materials, the layer-by-layer (LbL) assembly is a simple, versatile and well-established technique with high relevance in the drug delivery domain [1]. In fact, the LbL assembly, which entails the sequential adsorption of complementary species (mostly based on opposing charges) on a substrate, is enabling the creation of drug reservoirs either in the form of planar films or capsules with a high-level of control of drug administration, targeted delivery, and lower side effects. The presence of multiple layers in a single system facilitates the design of materials with a profusion of bioactive functionalities (e.g., antioxidant activity, antibacterial activity, and wound healing capacity) that are typical to their individual precursors [1].

Polysaccharides are amid the most commonly utilized components for the design of multi-layered systems applied on drug delivery, despite the constraint of not yielding robust and free-standing films or capsules without the use of a template, either sacrificial or non-sacrificial (e.g., glass slides, mesoporous silica, or calcium carbonate nanoparticles) [1]. In the context of planar films, bacterial cellulose (BC) membranes, viz. an exopolysaccharide produced by some non-pathogenic bacteria [2,3], can be used simultaneously as a non-sacrificial template and a drug reservoir. In fact, this robust and high purity cellulosic substrate, with a web-like entangled morphology, possesses in situ moldability that allows its direct biosynthesis in the form of membranes with personalized size and shape, revealing good in vitro biocompatibility and in vivo skin compatibility [4], alongside high water retention capacity, nanostructured porous network, high mechanical performance, and tailorable surface chemistry [5]. This set of intrinsic and unique features has been explored for the development of BC-based cutaneous drug delivery systems loaded with both hydrophilic or lipophilic active pharmaceutical ingredients (APIs) [6,7]. Nevertheless, the majority of the studies involves the simple diffusion of an API solution into the BC network followed by drying, which is a methodology that hinders the API selection and, in most instances, does not enable a controlled and targeted delivery of the APIs, particularly when they are highly water-soluble [7,8]. Herein, the use of the LbL assembly methodology, which can be performed in mild conditions in most substrates and enables the modulation of the composition, stability, and surface functionality of those substrates [1], can be a major asset for engineering BC-based membranes for wound healing applications, where a controlled and targeted drug delivery is an essential requirement.

Our interest in developing multi-layered BC derived patches by LbL assembly for cutaneous drug delivery originates from the idea of combining the peculiar properties of a freestanding exopolysaccharide robust membrane (i.e., BC) with the properties of hydrophilic ionic polysaccharides (i.e., chitosan (CH) and alginate (ALG)), and an API with wound healing potential (i.e., dexpanthenol (DEX) used in several pharmaceutical formulations in the field of dermatology and skin care). Although BC (either in its pure or oxidized forms) has already been individually blended with CH/ALG [9,10] or DEX [11], and BC nanocrystals have been used as a filler in an alginate matrix that was then covered with CH and gelatin polyelectrolytes via LbL assembly [12], there are no studies—as far as our literature search could verify—dedicated to the development of multi-layered patches composed of oxidized BC (OBC), CH, and ALG via spin-assisted LbL assembly technology, where the nanostructured porous cellulosic substrate plays the simultaneous role of a non-sacrificial template and a drug reservoir.

In this perspective, the goal of the present study is to design multi-layered patches composed of three polysaccharides, namely BC, CH, and ALG, as antibacterial cutaneous drug delivery vehicles for DEX, viz. a water-soluble API with wound healing ability. The multi-layered patches with a stratified structure were assembled via spin-assisted LbL methodology using a DEX-loaded OBC membrane as the initial negatively charged substrate, followed by the alternate adsorption of positively charged CH and negatively charged ALG polyelectrolytes, in a total of 5, 11, 17, and 21 layers. An in-depth analysis of the structure, morphology, thermal stability, mechanical properties, and moisture-uptake capacity of the multi-layered patches is presented, as well as the in vitro drug release profile at physiological pH, the in vitro antibacterial activity against *Staphylococcus aureus*, the in vitro cytotoxicity toward human HaCaT keratinocyte cells and the wound healing potential.

## 2. Materials and Methods

### 2.1. Chemicals, Bacteria, and Cells

Dexpanthenol (DEX, pharmaceutical secondary standard), glycerol (≥99.5%), phosphate buffered saline (PBS, pH 7.4), sodium hypochlorite solution (available chlorine 10–15%), sodium bromide (≥99%), sodium alginate (ALG, alginic acid sodium salt from brown algae, medium viscosity ≥2000 cP (2%, 25 °C)), 2,2,6,6-tetramethylpiperidine-1-oxyl (TEMPO free radical, >98%), tryptic soy broth (TSB), tryptic soy agar (TSA), 3-(4,5-dimethylthiazolyl-2)-2,5-diphenyltetrazolium bromide (MTT, 98%), and dimethyl sulfoxide (DMSO, ≥99.9%) were supplied by Sigma-Aldrich (Sintra, Portugal). Dulbecco′s Modified Eagle′s Medium (DMEM), fetal bovine serum (FBS), L-glutamine, penicillin/streptomycin, and fungizone were purchased from Gibco^®^ (Life Technologies, Carlsbad, CA, USA). Type 1 ultrapure water (resistivity of 18.2 MΩ cm (25 °C)) was filtered by a Simplicity^®^ Water Purification System (Merck Millipore, Darmstadt, Germany). Other chemicals and solvents were of laboratory grade.

Chitosan (CH from shrimp, degree of deacetylation of 98%, viscosity of 2900 cP (1% solution in 1% of acetic acid)) was obtained from Mahtani Chitosan Pvt. Ltd. India. For purification, CH was dissolved in a 1% (*v*/*v*) aqueous acetic acid solution, filtered, and precipitated by neutralization with NaOH 0.5 M up to a pH of 8.5. The precipitate was rinsed with water until neutral pH, followed by freeze-drying [13].

Bacterial cellulose (BC) was biosynthesized in our laboratory by the *Gluconacetobacter sacchari* bacterial strain in the form of wet membranes [14]. *Staphylococcus aureus* (ATCC 6538) bacterium was received from American Type Culture Collection (ATCC) collection (Manassas, VA, USA). The nontumorigenic immortalized human keratinocyte HaCaT cell line was acquired from Cell Lines Services (Eppelheim, Germany).

### 2.2. Preparation of Oxidized Bacterial Cellulose via TEMPO-Mediated Oxidation

The oxidized bacterial cellulose (OBC) membranes were prepared via TEMPO-mediated oxidation as described elsewhere, with some modifications [15]. Shortly, 50 g of never-dried BC were suspended into deionized water containing TEMPO (0.13 g) and NaBr (1.3 g) under magnetic stirring. Then, 0.267 g of NaClO aqueous solution was slowly added, and the pH of the solution was maintained at 10.5 by adding a 0.5 M NaOH aqueous solution. The temperature was kept at 30 °C throughout the reaction for 30 min. The oxidation was stopped by quenching with 40 mL of absolute ethanol, followed by washing with deionized water. The OBC membranes (carboxyl content of 0.54 ± 0.09 mmol g^−1^) were stored in distilled water at 4 °C until further use.

### 2.3. Preparation of Multi-Layered Patches Loaded with Dexpanthenol

First, ALG and CH solutions (1 mg mL^−1^) were prepared in ultrapure water and 1% (*v*/*v*) acid acetic aqueous solution, respectively. Wet OBC membranes (6 × 4 cm^2^) were weighted, drained, and then soaked in 5 mL of an aqueous buffered solution (pH 7.4) of DEX (0.15% *w*/*v*) with glycerol (1% *w*/*v*). The membranes were stirred at 100 rpm and 30 °C for 1 h to allow the complete absorption of the solution (viz. 100% entrapment efficiency) and then left to dry at 30 °C for 24 h. The DEX-loaded OBC patch was coated with 250 μL of CH (zeta (ζ)-potential: 57 ± 3 mV (Zetasizer Nano ZS, Malvern Panalytical, Cambridge, United Kingdom)) and 250 μL of ALG (ζ-potential: −40 ± 3 mV) aqueous solutions by spin coating (Spin 150, APT GmbH–Automation und Produktionstechnik, Germany) at 2000 rpm during 15 s. A total of 5, 11, 17, or 21 layers of the polysaccharides were deposited, starting and finishing with CH, as compiled in Table 1.

### 2.4. Characterization Techniques

The thickness of the specimens was measured with a Mitutoyo coolant-proof digimatic micrometer MDC-25PX (Mitutoyo Corporation, Tokyo, Japan). All measurements were executed at fifteen different locations of the specimens, randomly selected, with an accuracy of 1 μm.

Attenuated total reflection-Fourier transform infrared (ATR-FTIR) spectra were computed on a Perkin-Elmer FT-IR System Spectrum BX spectrophotometer (Perkin-Elmer Inc., Waltham, MA, USA) coupled with a single horizontal Golden Gate ATR cell (Specac^®^, London, UK), in the wavenumber range of 600–4000 cm^−1^ at a resolution of 4 cm^−1^ over 32 scans.

Scanning electron micrographs (SEM) were recorded in a HR-FESEM SU-70 Hitachi microscope (Hitachi High-Technologies Corporation, Tokyo, Japan). The specimens were placed on an aluminum plate and formerly coated with a carbon film.

Thermogravimetric analysis (TGA) was undertaken in a SETSYS SETARAM TGA analyzer (SETARAM Instrumentation, Lyon, France). The specimens were heated from 25 °C to 800 °C at a heating rate of 10 °C min^−1^ under a nitrogen atmosphere.

Tensile mechanical tests were conducted on a uniaxial Instron 5966 testing apparatus (Instron Corp., Rockville, MD, USA) in traction mode at a deformation rate of 10 mm min^−1^ using a 500 N static load cell and gauge length of 30 mm. The specimens were rectangular strips (5 × 1 cm^2^) dried at 40 °C. All measurements were held on seven replicates.

The moisture-uptake capacity was assessed by laying the dry specimens (2 × 2 cm^2^) in a conditioned cabinet with 100% relative humidity at 25 °C for 22 h. After that period, the weight (*W_w_*) of the moist specimens was measured, and the moisture-uptake was determined according to the equation:

(1) $$\mathrm{Moisture}-\mathrm{uptake}\;\left(\%\right)=\left(W_{w}-W_{0}\right)\times W_{0}^{-1}\times 100$$

where *W*_0_ is the initial weight of the dry specimens [16].

### 2.5. In Vitro Drug Release Assays

DEX-loaded patches (2 × 2 cm^2^) were immersed in a vessel containing 30 mL of 0.01 M PBS (pH 7.4) and the dissolution tests were then carried out at 32 °C and 50 rpm during 90 h. At regular time intervals, aliquots of 3 mL of each solution were withdrawn, and the same volume of fresh PBS was added to maintain a constant volume. The DEX concentration in each aliquot was determined by UV–Vis spectroscopy (Shimadzu UV-1800 UV–Vis spectrophotometer, Shimadzu Corp., Kyoto, Japan) at 206 nm with the linear calibration curve: y=0.135x+0.0167 (R^2^ = 0.9978), in the range of 10–100 µm mL^−1^. The DEX content at each time was plotted as a cumulative percentage release concentration calculated using the formula:

(2) $$C_{\mathrm{cumulative}}=C_{n}+\left[\left(3\times C_{n-1}\right)/30\right]$$

where *C_n_* and *C_n_*_−1_ are the concentrations of DEX at time *n* and *n* − 1, respectively. Nine replicates were performed for each specimen [8].

### 2.6. In Vitro Antibacterial Activity

The antibacterial activity of the OBC-based membranes was examined against *S. aureus*. The bacterial pre-inoculum cultures were grown for 24 h in TSB growth medium at 37 °C under horizontal shaking at 120 rpm until reaching a concentration of 10^8^ to 10^9^ colony forming units per mL (*CFU* mL^−1^). Each film specimen (4 × 6 cm^2^) was placed in contact with 5 mL of bacterial suspension via a ten-fold dilution of the overnight grown culture in PBS (pH 7.4). A bacterial cell suspension was tested as the control while OBC membranes, neat and DEX-loaded, were tested as blank references. All specimens were incubated at 37 °C under horizontal shaking at 120 rpm. At 24 h contact time, aliquots (500 μL) of each specimen and controls were collected and the bacterial concentration (*CFU* mL^−1^) was determined by plating serial dilutions on TSA medium. The plates were incubated at 37 °C for 48 h. The *CFU* were determined on the most appropriate dilution on the agar plates. Two independent experiments were carried out and, for each, two replicates were plated. The bacteria log reduction of the specimens was calculated as follows:

(3) $$\log\;CFU\;{\mathrm{mL}}^{-1}reduction=\log CFU_{control}-\log CFU_{patch}$$

### 2.7. In Vitro Cytotoxicity Assay

The cytotoxicity of the patches was evaluated in human keratinocytes cell line (HaCaT cells) by the MTT assay [17]. Briefly, cells were grown in complete DMEM medium supplemented with 10% FBS, 2 mM L-glutamine, 10,000 U mL^−1^ penicillin/streptomycin, and 250 µg mL^−1^ fungizone at 37 °C in 5% CO_2_ humidified atmosphere. Cells were observed daily under an inverted phase-contrast Eclipse TS100 microscope (Nikon, Tokyo, Japan). The tests were performed in triplicates of OBC, OBC/DEX, and OBC/DEX/CH/ALG_21. Specimens of 1 × 1 cm² were prepared, sterilized by ultraviolet radiation, and then incubated with 1.0 mL of complete DMEM medium at 37 °C with 5% CO_2_ for 24 h to prepare the specimen extract.

Meanwhile, one 96-well plate was prepared with 5 × 4 wells filled up with 6000 cells/well and the cells were then incubated in complete culture medium for 24 h for adhesion. After that time, cell culture medium (in the 96-well plates) was substituted by 100 μL of each of the extracts obtained from the incubated specimens and cells were then additionally incubated for either 24 h or 48 h. As a negative control, HaCaT cells were incubated with complete DMEM medium and treated identically as described for the specimens.

At the end of the incubation time, 50 μL of MTT (at a concentration of 1 g L^−1^) was added to each well and incubated for 4 h at 37 °C in a 5% CO_2_ humidified atmosphere. After that, the culture medium with MTT was removed and replaced by 150 μL of DMSO and the plate was positioned in a shaker for 2 h in the dark to totally dissolve the formazan crystals. The absorbance of the specimens was measured with a BioTek Synergy HT plate reader (Synergy HT Multi-Mode, BioTeK, Winooski, VT, USA) at 570 nm with blank corrections. The cell viability was calculated with respect to the control cells:

(4) $$\mathrm{Cell}\;\mathrm{viability}\;\left(\%\right)=\left[\left(Abs_{sample}-Abs_{DMSO}\right)/\left(Abs_{control}-Abs_{DMSO}\right)\right]\times 100$$

where the $Abs_{sample}$ is the absorbance of the specimen; $Abs_{DMSO}$ is the absorbance of the *DMSO* solvent; and $Abs_{control}$ is the absorbance of the control.

### 2.8. In Vitro Wound Healing (Scratch) Assay

The effect of the multi-layered patches on the migration capability of HaCaT keratinocyte cells was evaluated using the scratch assay. Cells were seeded in a 6-well plate at 5 × 10^5^ cells/well and the linear wound was generated with a sterile 200 μL plastic pipette tip. Specimens of 1 × 1 cm² were prepared, sterilized by ultraviolet radiation, and then incubated with 1.0 mL of complete DMEM medium at 37 °C, with 5% CO_2_ for 24 h to prepare the specimen extract. The tests were performed in triplicates of OBC/DEX, OBC/DEX/CH/ALG_11, and OBC/DEX/CH/ALG_21. As a negative control, HaCaT cells were treated identically as described for the specimens. Each specimen extract was applied to the scratch and the cells were then further incubated for 9 and 24 h at 37 °C, with 5% CO_2_. Optical micrographs (Eclipse TS100 microscope, Nikon, Tokyo, Japan) of the scratched area of each condition were taken and compared to the corresponding 0 h time point.

### 2.9. Statistical Analysis

The statistical analysis was conducted with OriginPro 9.0.0 (OriginLab Corporation, Northampton, MA, USA), where the statistical significance was assessed by the analysis of variance (ANOVA) and Tukey’s test with the statistical significance established at *p* < 0.05.

## 3. Results and Discussion

**Figure 1:**
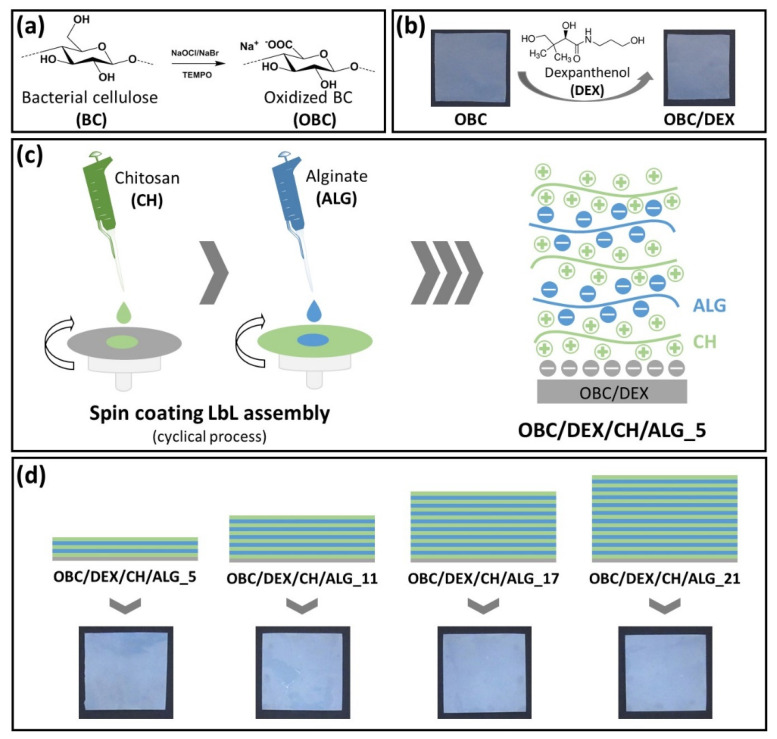
Schematic representation of (**a**) TEMPO-mediated oxidation of BC yielding OBC, (**b**) loading of OBC with DEX, (**c**) spin coating LbL assembly of the polysaccharides multi-layered patch with 5 layers, and (**d**) digital photographs of the OBC/DEX/CH/ALG patches with 5, 11, 17, and 21 layers.

Polysaccharides-based multi-layered patches composed of OBC, CH, and ALG were fabricated by spin-assisted LbL coating assembly technology, as illustrated in Figure 1. This assembly methodology was selected due to its simplicity and the stratified structure it conveys to the materials [1]. Therefore, the first step to prepare the multi-layered membranes comprised the functionalization of BC via the environmentally friendly TEMPO-mediated oxidation to obtain oxidized bacterial cellulose membranes with negatively charged groups at the surface (Figure 1a). The water-soluble radical reagent TEMPO is a catalytic oxidant that jointly with NaBr and NaClO selectively converts the cellulose C6 hydroxyl groups into carboxylic moieties in aqueous media, without compromising the morphological integrity of the fibers [18].

The OBC membrane was then loaded with DEX (i.e., OBC/DEX membrane, Figure 1b), which is a water-soluble active pharmaceutical ingredient (API) used in several pharmaceutical formulations in the field of dermatology and skin care due to its capacity to act as a skin moisturizer, skin barrier restorer, and facilitator of wound healing [19]. All membranes contain 0.32 mg of DEX per cm^2^ of OBC, selected based on equivalent commercial formulations [19]. Further to this, glycerol (1% *w*/*v*, 2.1 mg per cm^2^ of membrane) was incorporated in the membrane as a plasticizer to upsurge their malleability and conformability to the skin surface [20].

Afterward, four multi-layered patches with a distinct number of layers (5, 11, 17, and 21) of the positively charged CH (ζ-potential: 57 ± 3 mV) and the negatively charged ALG (ζ-potential: −40 ± 3 mV) polyelectrolytes were prepared (Figure 1c). CH and ALG polysaccharides were selected as the oppositely charged polyelectrolytes because of their efficient film-forming ability and vast applicability in the LbL assembly technology [1]. Furthermore, CH was chosen as the final layer in the four multi-layered membranes due to its known antibacterial activity toward a wide variety of microorganisms [13,21], largely linked to the presence of amino groups [22]. This bioactive property is particularly pertinent in the context of skin regeneration to avoid the growth of pathogenic microorganisms that will contribute to microbial wound infections [23,24].

All multi-layered patches are whitish and homogeneous, as displayed in the photographs of Figure 1d, confirming the even distribution of DEX and of the polyelectrolytes (i.e., CH and ALG). Predictably, the weight and thickness of the multi-layered patches increased with the increasing number of layers as listed Table 1. The nanostructured patches were characterized in terms of structure by ATR-FTIR spectroscopy, morphology by SEM, thermal stability by TGA, mechanical properties by tensile tests and moisture-uptake capacity. Additionally, the in vitro drug release profile, in vitro antibacterial activity, in vitro cytotoxicity, and in vitro wound healing potential were also evaluated.

### 3.1. Structural and Morphological Characterization

**Figure 2:**
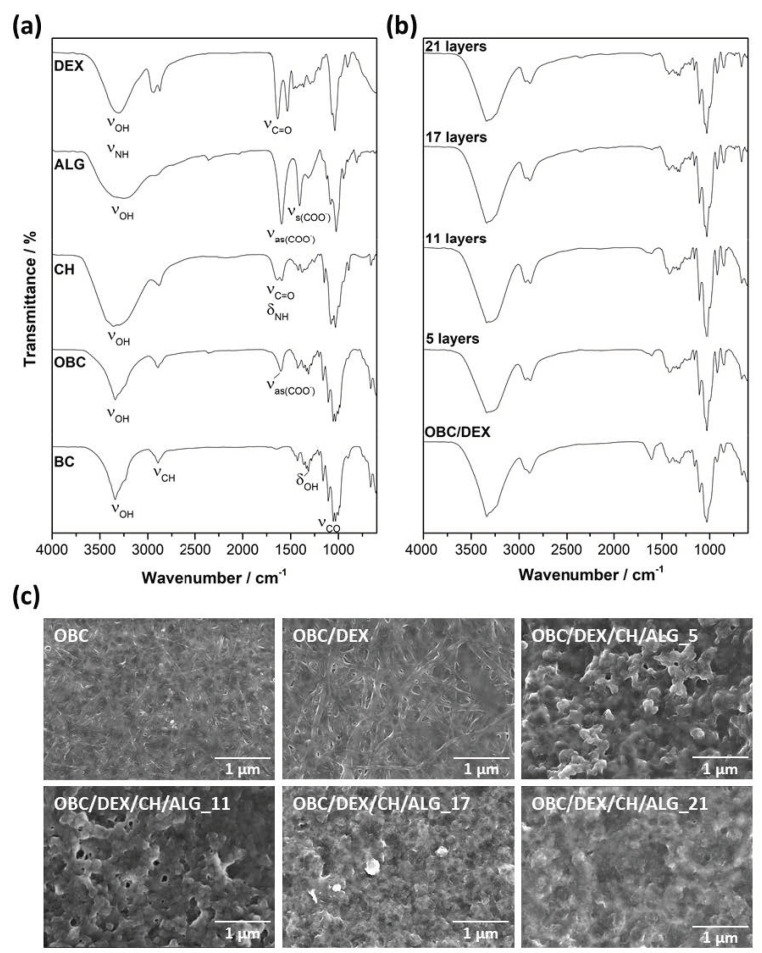
(**a**,**b**) ATR-FTIR spectra (vibrational modes: *ν* = stretching, *δ* = bending) of BC, OBC, CH, ALG, DEX (**a**), OBC/DEX, and OBC/DEX/CH/ALG-based multi-layered patches with 5, 11, 17, and 21 layers (**b**), and (**c**) SEM surface micrographs (×30.0 k magnification) of OBC, OBC/DEX, and OBC/DEX/CH/ALG-based multi-layered patches with 5, 11, 17, and 21 layers.

The modification of BC via TEMPO-mediated oxidation to obtain OBC with negatively charged groups was confirmed by ATR-FTIR spectroscopy through the presence of the typical absorption bands of the BC backbone at 1029 cm^−1^ (C–O stretching), 1316 cm^−1^ (O–H in plane bending), 2897 cm^−1^ (C–H stretching), and 3340 cm^−1^ (O–H stretching) [25], and the emergence of the absorption band at 1590–1640 cm^−1^ assigned to the antisymmetric vibration of ionized carboxyl groups (ν_as_(COO^–^), Figure 2a) [26]. Moreover, the SEM micrograph of the OBC membrane surface (Figure 2c) shows the characteristic three-dimensional nanofibrillar structure of BC [5,14]. This means that the oxidation of BC did not affect the morphology (Figure 2c) nor the visual appearance of the membranes.

The incorporation of DEX via diffusion into the three-dimensional porous network of OBC was also validated by ATR-FTIR spectroscopy (Figure 2b) through the presence of the typical absorption bands of each component (Figure 2a), whilst the ones allocated to DEX, namely at 3308 cm^−1^ (O–H and N–H stretching), 1636 cm^−1^ (C=O stretching, amide I band), and 1535 cm^−1^ (amide II band) [26,27] are not completely observable given the small amount of API present in the patch (0.32 mg of DEX per cm^2^ of OBC). In the SEM micrograph of the surface of the OBC/DEX patch (Figure 2c), the nanofibrillar structure of OBC is still visible, suggesting that all DEX was absorbed into the three-dimensional porous network of OBC and none remains at the surface of the patch, confirming the above vibrational spectroscopy data.

The ATR-FTIR spectra of the multi-layered patches (Figure 2b) portrayed similarities with those of their precursors (Figure 2a), mainly with OBC, but also with chitosan at 3342 cm^−1^ (O–H stretching), 1638 cm^−1^ (C=O stretching and N−H bending), 1592 cm^−1^ (−NH_2_ bending), 1380 cm^−1^ (−CH_2_ bending), 1148 cm^−1^ (C−O−C antisymmetric stretching), 1078 and 1032 cm^−1^ (C−O stretching) [28], and ALG at 3250 cm^−1^ (O–H stretching), and 1596 cm^−1^ (COO^−^ antisymmetric stretching) and 1406 cm^−1^ (COO^−^ symmetric stretching) [29]. Moreover, the absorption bands of glycerol at 1100 cm^−1^ and 1030 cm^−1^ (C–O stretching vibrations of alcohols) and 3250 cm^−1^ (O–H stretching), [7] are fully superimposed with the vibrations of the polysaccharides, viz. OBC, CH, and ALG.

Regarding the morphology (Figure 2c), the SEM micrographs of the surface of the multi-layered membranes did not disclose the three-dimensional fibrillar structure of OBC because it was completely covered by the various CH and ALG layers. Furthermore, the micrographs also depicted the formation of micron-size particles credited to the polyelectrolytes, which underlines the homogeneous and total coverage of the negatively charged OBC substrate. Indeed, in the case of the patch with 21 layers, the micrograph resembles the formation of a particulate film.

### 3.2. Thermal Stability and Mechanical Properties

**Figure 3:**
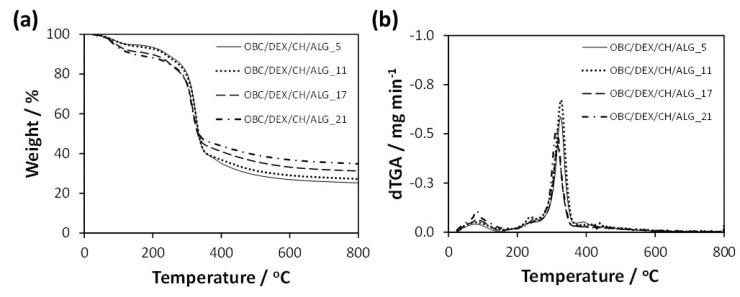
(**a**) Thermograms and (**b**) derivative curves of the multi-layered patches: OBC/DEX/CH/ALG_5, OBC/DEX/CH/ALG_11, OBC/DEX/CH/ALG_17, and OBC/DEX/CH/ALG_21.

The thermal stability of the multi-layered patches was measured by thermogravimetric analysis under a nitrogen atmosphere. According to Figure 3, the data did not vary appreciably with the increasing number of polyelectrolyte layers (i.e., 5, 11, 17, and 21 layers), with all membranes exhibiting a double weight-loss degradation profile with thermal stability up to about 200 °C (Figure 3b). Apart from the initial volatilization of water at ca. 100 °C, the small first step with a maximum rate of decomposition temperature at ca. 240 °C is allocated to the volatilization of glycerol [30] and degradation of DEX [27], while the second step with a maximum rate of decomposition temperature in the range 320–330 °C is related to the polysaccharide skeleton degradation, namely OBC [31], CH [13], and ALG [32]. In this second-step, the maximum degradation temperature slightly decreases with the increasing number of layers, most likely due to the marginally lower thermal stability of the polyelectrolytes [13,32]. Furthermore, the final residue of the multi-layered patches at 800 °C varied between 25% for OBC/DEX/CH/ALG_5 and 35% for OBC/DEX/CH/ALG_21, which is mainly attributed to the increase of CH and ALG in the membranes. However, it is worth remarking that all multi-layered OBC/DEX/CH/ALG-based membranes are thermally stable up to 200 °C, and therefore, can undergo the sterilization procedures (e.g., autoclaving at ca. 120 °C) required for biomedical applications.

The mechanical properties of the multi-layered polysaccharides-based patches were also analyzed, and the data obtained from the stress-strain curves are condensed in Table 2. The negatively charged OBC substrate exhibits an elongation at break of 3.4 ± 0.6%, tensile strength of 150 ± 25 MPa and Young’s modulus of 8.2 ± 1.4 GPa. Furthermore, the inclusion of DEX (dissolved in a buffered solution with glycerol (1% *w*/*v*)) into the OBC porous structure promoted a decrease in both Young’s modulus and tensile strength to 3.5 ± 1.2 GPa and 97 ± 15 MPa, respectively, complemented by an increase in the elongation at break to 4.9 ± 0.6%. This behavior is comparable to the trend obtained for pure BC membranes after the incorporation of APIs such as diclofenac [33] and lidocaine [34] into the BC three-dimensional structure. Furthermore, Tanrıverdi et al. also reported a decrease in the tensile strength of synthetic nanofiber mats, namely poly(lactic-*co*-glycolic acid), poly(ethylene oxide), and poly(ɛ-caprolactone), loaded with DEX [35].

After the LbL assembly of the oppositely charged polyelectrolytes (i.e., CH and ALG) on the negatively charged OBC substrate, the mechanical performance of the multi-layered membranes increased with the increasing number of layers. The Young’s modulus increased from 4.2 ± 0.8 GPa for OBC/DEX/CH/ALG_5 to 10.9 ± 4.0 GPa for OBC/DEX/CH/ALG_21, whereas the tensile strength increased from 142 ± 39 MPa for OBC/DEX/CH/ALG_5 to 188 ± 57 MPa for OBC/DEX/CH/ALG_21. On the contrary, the elongation at break decreased from 4.3 ± 0.7% for OBC/DEX/CH/ALG_5 to 2.2 ± 0.4% for OBC/DEX/CH/ALG_21, meaning that the membranes became less elastic as the number of layers increased from 5 to 21. Nonetheless, these hydrophilic multi-layered patches are satisfactorily malleable to conform to the surface of the skin.

Worth noting is the fact that these OBC/DEX/CH/ALG multi-layered patches exhibited a higher tensile strength than the similar non-multi-layered films based on hydrogen peroxide oxidized BC (HOBC) or periodic acid oxidized BC (POBC) containing CH and ALG, which were prepared by blending the homogenized HOBC or POBC pulp with a CH and ALG gel solution, and studied for application as wound dressings [10].

### 3.3. Moisture-Uptake Capacity

**Figure 4:**
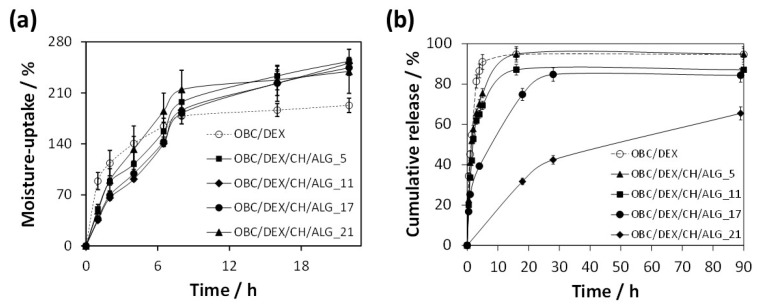
(**a**) Moisture-uptake capacity and (**b**) DEX cumulative release profile of OBC, OBC-loaded with DEX, and multi-layered polysaccharides-based patches.

The moisture-uptake capacity of the multi-layered patches was determined to anticipate their interaction with moisture and exudates when applied to wound sites. Hence, the patches were left in a chamber with 100% relative humidity at 25 °C for 22 h. According to Figure 4, all OBC/DEX/CH/ALG patches absorbed moisture over time and were able to reach values of 240–250% after 22 h of exposure. Furthermore, the time-dependent data did not vary appreciably with the increasing number of layers (i.e., 5, 11, 17, and 21 layers), meaning that these patches present an analogous moisture-uptake capacity. This behavior is obviously credited to the hydrophilic nature of the precursors of the OBC/DEX/CH/ALG-based membranes, as in fact shown for the non-multi-layered films composed of OBC, CH, and ALG [10].

Therefore, any of these multi-layered polysaccharide-based patches can promote (if necessary) a moist environment at the skin interface and remove the surplus of exudates or other body fluids when applied to the skin surface.

### 3.4. In Vitro Drug Release Profile

The in vitro release profile of the multi-layered polysaccharides-based patches was tested in PBS (pH 7.4) at 32 °C that simulates the usual pH and temperature of the skin [36]. Figure 4b shows the cumulative release profiles of DEX from the OBC/DEX/CH/ALG-based membranes, which were compared with the in vitro release of DEX from a membrane composed solely of OBC and DEX.

In general, the release profile of the patches is quite standard, with an initial burst followed by a plateau where the DEX release achieves the highest value. While the OBC/DEX and OBC/DEX/CH/ALG_5 patches reached a maximum cumulative release of ca. 95% after 16 h, the OBC/DEX/CH/ALG_11 achieved ca. 87% after 16 h and OBC/DEX/CH/ALG_17 attained ca. 85% after 28 h. In the case of the OBC/DEX/CH/ALG_21 patch, it only reached a maximum cumulative release of ca. 65% after 90 h. This is a direct indication that the multi-layered systems retarded the DEX release from the OBC membrane, particularly for 21 layers. Moreover, this slow-release profile is suited for wounds that require longer therapeutic treatments combined with a local skin moisturizer with antibacterial properties and wound healing ability.

When compared with non-multi-layered BC-based membranes loaded with water-soluble APIs [7,8], these OBC/DEX/CH/ALG multi-layered patches presented a controlled release profile that only reached high cumulative release values after several hours.

The in vitro drug release curves (Figure 4b) were further adjusted to kinetic models to identify the diffusional release mechanism of DEX from the multi-layered OBC/DEX/CH/ALG-based membranes. The data were better fitted to the Korsmeyer–Peppas kinetic model [37,38], $M_{t}/M_{\infty }=kt^{n}$, with $M_{t}$ as the DEX content released at time t, $M_{\infty }$ is the DEX content released at infinite time, k is the kinetic constant, and n is the diffusion constant denoting the release mechanism [37,39]. Based on this kinetic model, only the cumulative release values of $M_{t}/M_{\infty }<60\%$ are fitted, and therefore, a release exponent (*n*) of 0.45 was obtained for OBC/DEX, 0.68 for OBC/DEX/CH/ALG_5, 0.69 for OBC/DEX/CH/ALG_11, 0.68 for OBC/DEX/CH/ALG_17, and 0.70 for OBC/DEX/CH/ALG_21 (Table 3). These curve fitting parameters are representative of Fickian diffusion (i.e., diffusion-controlled drug release mechanism, in the case of OBC/DEX, and non-Fickian transport (0.5 < *n* < 1.0) (i.e., diffusion and swelling controlled drug release mechanism), for the case of the multi-layered polysaccharide-based membranes [37,38,39].

### 3.5. In Vitro Antibacterial Activity

**Figure 5:**
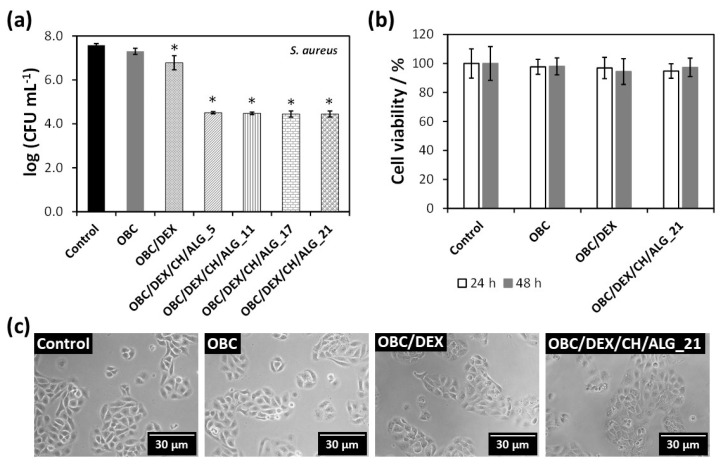
(**a**) Antibacterial activity (24 h of exposure) of OBC, OBC-loaded with DEX, and multi-layered patches (the symbol * represents the means with a significant difference (*p* < 0.05) from the control and OBC); (**b**) cell viability of HaCaT cells after 24 h and 48 h of exposure to negative control, OBC, OBC/DEX, and OBC/DEX/CH/ALG_21, and (**c**) the corresponding optical micrographs of HaCaT cells after 24 h of exposure.

The antibacterial activity of the multi-layered polysaccharide-based patches was assayed against *S. aureus* (a Gram-positive bacterium) for 24 h, as shown in Figure 5a. This bacterium has been extensively studied because it is the culprit of several skin infections, particularly in patients with skin injuries [23]. As expected, neither the control nor the OBC membrane hindered the growth of the Gram-positive pathogenic microorganism. This observation is consistent with the literature, since OBC does not inhibit the growth of *S. aureus*, as previously reported [40]. Nevertheless, when DEX is loaded into the OBC membrane, the bacterial growth of *S. aureus* was reduced by 0.8–log *CFU* mL^–1^ after 24 h. Although DEX is not an antibacterial agent [41], it seems that its combination with OBC conveyed a small growth inhibition to the OBC/DEX membrane.

Regarding the multi-layered polysaccharide-based patches, they are responsible for ca. 3.2–log *CFU* mL^–1^ reduction after 24 h (Figure 5a). Since the general tendency of the four membranes was the same, it is fair to claim that all OBC/DEX/CH/ALG patches are antibacterial agents due to the presence of chitosan. In fact, several studies have shown that the marine polysaccharide derived from chitin (i.e., chitosan) presents antibacterial activity toward a wide variety of microorganisms [21] including *S. aureus* [13], largely linked to the presence of amino groups [22,24]. This bioactive property is of paramount importance for skin regeneration to avert the growth of pathogenic microorganisms causing microbial wound infections [23,24].

### 3.6. In Vitro Cytotoxicity Assays

The cytotoxic behavior of the multi-layered patch with 21 layers (OBC/DEX/CH/ALG_21) as well as that of OBC and OBC/DEX was assessed in human HaCaT keratinocyte cells via the indirect MTT assay. This cell line was elected because it has been intensely used as a model for epidermal cells [42,43,44,45]. Figure 5b shows that the HaCaT cells metabolic activity was normal for the negative control (100% cell viability) as well as for the OBC membrane (ca. 98%) for both exposure times (24 and 48 h). Therefore, the OBC membrane is non-cytotoxic toward HaCaT cells, which is coherent with the results achieved in the literature for other cell lines such as NIH3T3 fibroblast cells [31]. Furthermore, the cell viability of the OBC patch loaded with DEX was almost unaltered with values of 97 ± 7% after 24 h and 94 ± 9% after 48 h. This behavior was predictable because DEX is a non-cytotoxic drug used in several pharmaceutical formulations in the field of dermatology and skin care [19].

With regard to the multi-layered patch with the higher number of layers (i.e., OBC/DEX/CH/ALG_21), it was also deemed as a non-cytotoxic material because the cell viability was 95 ± 5% after 24 h and 97 ± 6% after 48 h (Figure 5b). Furthermore, the optical micrographs of the HaCaT cells (Figure 5c) support the cell viability data by evidencing that neither the cell morphology nor the cell count varied in relation to the control after 24 h of cell incubation with the OBC, OBC/DEX, and OBC/DEX/CH/ALG_21 membranes. Actually, all values were high above the 70% threshold of cell viability [46], which reflects the potential in vivo performance of these safe and compatible multi-layered patches for application in the biomedical field.

### 3.7. In Vitro Wound Healing Activity

**Figure 6:**
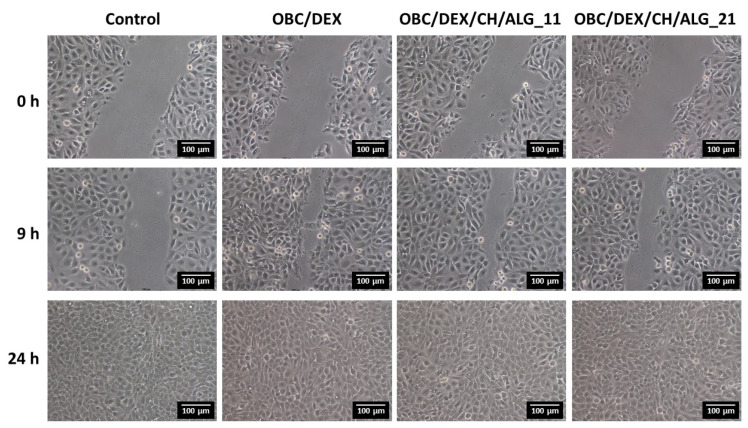
Optical micrographs of the scratch assay of the HaCaT cells after 9 and 24 h of exposure to control, OBC/DEX, OBC/DEX/CH/ALG_11, and OBC/DEX/CH/ALG_21 patches.

Having proved that the multi-layered polysaccharide-based patches are robust, thermally stable, and present a controlled drug release profile, antibacterial activity against *S. aureus* and non-cytotoxicity toward HaCaT cells, the next step focused on the wound healing potential of these patches as delivery vehicles for DEX. Therefore, the wound healing activity of the multi-layered polysaccharide-based patches with 11 and 21 layers (OBC/DEX/CH/ALG_11 and OBC/DEX/CH/ALG_21) as well as of the OBC/DEX patch was assessed using the in vitro wound healing scratch assay [47]. Therefore, the influence of the patches on the cell migration was studied for HaCaT keratinocyte cells after 9 and 24 h of exposure. Predictably, and as shown in Figure 6, the cells cultivated in a medium containing only culture medium (i.e., control) presented a good migration capacity with the complete occlusion of the scratch after 24 h.

Overall, and in the case of the patches, the HaCaT keratinocyte cells situated near the boundary of the scratched region moved toward the vacant region as time progressed, achieving a full closure after 24 h. Furthermore, the cell density at the back of the scratch site is also growing with time, which points to the proliferation of the HaCaT keratinocyte cells. However, in terms of temporal progression, the patches containing DEX, namely OBC/DEX, OBC/DEX/CH/ALG_11 and OBC/DEX/CH/ALG_21, demonstrated higher coverage of the wound-like gap area after 9 h, induced by cell growth and migration, when compared to the positive control. Despite the analogous behavior of the HaCaT keratinocyte cells after 24 h of exposure to the three patches (Figure 6), the optical micrographs after 9 h show a slightly slower cell growth and migration for the patches with 11 and 21 layers. In fact, the behavior of the different patches after 9 h of exposure (Figure 6) seems to be in line with the in vitro drug release profile of DEX, where the OBC/DEX patch is the one releasing the higher amount of DEX in the shorter period of time and the multi-layered OBC/DEX/CH/ALG_21 membrane with a controlled drug release profile is the one releasing the lower content of DEX after 24 h, as depicted in Figure 4b. Similar observations in terms of wound closure as a function of time were documented by Sharma et al. [48] for curcumin conjugated with hyaluronic acid, which are two natural compounds known to expedite the wound healing process, and by Silva et al. [16] for multifunctional nanofibrous patches composed of nanocellulose and lysozyme nanofibers.

Given that wound closure is a signal of wound healing ability [49], these antibacterial and non-cytotoxic multi-layered polysaccharide-based patches have the potential to foster wound healing and, simultaneously, hinder skin infections. Moreover, the drug release profile can be tuned to the specific type of wound and required treatment time by simply adjusting the number of polysaccharide layers.

## 4. Conclusions

Functional multi-layered patches composed of OBC, CH, ALG, and DEX were prepared for wound healing applications. Four multi-layered patches with different number of layers, viz. 5, 11, 17, and 21, were assembled by the spin assisted LbL coating technique of oppositely charged CH and ALG polyelectrolytes onto the OBC cellulosic substrate loaded with DEX. These patches with a stratified structure had thermal stability up to almost 200 °C, mechanical properties with a Young’s modulus higher than 4 GPa, and good moisture-uptake capacity (240–250%). Additionally, they inhibited the growth of *Staphylococcus aureus* with 3.2–log *CFU* mL^–1^ reduction and are non-cytotoxic to human keratinocytes (HaCaT cells) with a cell viability of about 97% after 48 h. The in vitro DEX release profile from the patches is time-dependent and is prolonged with the increasing number of layers, and the in vitro wound healing assay showed a good migration capacity with the full occlusion of the wound scratch after 24 h. These data validate the potentiality of these antibacterial multi-layered polysaccharides-based patches as vehicles for the topical delivery of DEX, which is a promoter of wound healing.

## Figures and Tables

**Table 1:** List of patches with the respective dry weight and thickness values.

Patch ^a^	Layers	Dry weight/mg ^b^	Thickness/µm ^b^
OBC	OBC	267 ± 10	45 ± 10
OBC/DEX	OBC(DEX)	275 ± 11	50 ± 15
OBC/DEX/CH/ALG_5	OBC(DEX)-CH-ALG-CH-ALG-CH	277 ± 13	55 ± 10
OBC/DEX/CH/ALG_11	OBC(DEX)-CH-ALG-CH-ALG-CH-ALG-CH-ALG-CH-ALG-CH	279 ± 13	61 ± 11
OBC/DEX/CH/ALG_17	OBC(DEX)-CH-ALG-CH-ALG-CH-ALG-CH-ALG-CH-ALG-CH-ALG-CH-ALG-CH-ALG-CH	284 ± 10	65 ± 16
OBC/DEX/CH/ALG_21	OBC(DEX)-CH-ALG-CH-ALG-CH-ALG-CH-ALG-CH-ALG-CH-ALG-CH-ALG-CH-ALG-CH-ALG-CH-ALG-CH	286 ± 11	69 ± 13

^a^ All patches contain glycerol as a plasticizer (1% *w*/*v*, 2.1 mg per cm^2^ of patch) that was incorporated inside the OBC; ^b^ Values are expressed as mean ± standard deviation.

**Table 2:** Young’s modulus, tensile strength, and elongation at break of the OBC, OBC/DEX, and the multi-layered patches.

Patch ^a^	Young’s Modulus/GPa	Tensile Strength/MPa	Elongation at Break/%
OBC	8.2 ± 1.4	150 ± 25	3.4 ± 0.6
OBC/DEX	3.5 ± 1.2	97 ± 15	4.9 ± 0.6
OBC/DEX/CH/ALG_5	4.2 ± 0.8	142 ± 39	4.3 ± 0.7
OBC/DEX/CH/ALG_11	4.4 ± 0.8	154 ± 52	4.0 ± 1.1
OBC/DEX/CH/ALG_17	9.8 ± 2.7	166 ± 45	2.0 ± 0.4
OBC/DEX/CH/ALG_21	10.9 ± 4.0	188 ± 57	2.2 ± 0.4

^a^ All patches contain glycerol as a plasticizer.

**Table 3:** Fitting parameters of the Korsmeyer–Peppas kinetic model and the corresponding drug release mechanism.

Patch	Release Exponent (*n*)	Regression Coefficient (R^2^)	Drug Release Mechanism
OBC/DEX	0.45	0.997	Fickian
OBC/DEX/CH/ALG_5	0.68	0.998	Non-Fickian
OBC/DEX/CH/ALG_11	0.69	0.997	Non-Fickian
OBC/DEX/CH/ALG_17	0.68	0.998	Non-Fickian
OBC/DEX/CH/ALG_21	0.70	0.999	Non-Fickian
